# Experimental Characterization and Analysis of the In-Plane Elastic Properties and Interlaminar Fracture Toughness of a 3D-Printed Continuous Carbon Fiber-Reinforced Composite

**DOI:** 10.3390/polym14030506

**Published:** 2022-01-27

**Authors:** Jonnathan D. Santos, Alex Fernández, Lluís Ripoll, Norbert Blanco

**Affiliations:** 1 Grupo de Investigación en Nuevos Materiales y Procesos de Transformación (GIMAT), Universidad Politécnica Salesiana, Calle Vieja 12-30 y Elia Liut, Cuenca 010105, Ecuador; jsantos@ups.edu.ec; 2 Analysis and Advanced Materials for Structural Design (AMADE), Department of Mechanical Engineering and Industrial Construction, Universitat de Girona, Avda. M. Aurèlia Capmany 61, 17003 Girona, Spain; alex.fernandez@udg.edu (A.F.); lluis.ripoll@udg.edu (L.R.)

**Keywords:** 3D-printed composite, interlaminar fracture toughness, experimental characterization

## Abstract

The use of continuous fiber as reinforcement in polymer additive manufacturing technologies enhances the mechanical performance of the manufactured parts. This is the case of the Carbon-Fiber reinforced PolyAmide (CF/PA) used by the MarkForged MarkTwo^®^ 3D printer. However, the information available on the mechanical properties of this material is limited and with large variability. In this work, the in-plane mechanical properties and the interlaminar fracture toughness in modes I and II of Markforged’s CF/PA are experimentally investigated. Two different standard specimens and end-tabs are considered for the in-plane properties. Monolithic CF/PA specimens without any additional reinforcement are used for the interlaminar fracture toughness characterization. Two different mode I specimen configurations are compared, and two different test types are considered for mode II. The results show that prismatic specimens with paper end-tabs are more appropriate for the characterization of the in-plane material properties. The use of thick specimens for mode I fracture toughness tests complicates the characterization and can lead to erroneous results. Contrary to what has been reported in the literature for the same material, fracture toughness in mode I is lower than for mode II, which agrees with the normal tendency of traditional composite materials.

## 1. Introduction

The superior properties of fiber composites are well known, especially their high strength-to-weight and stiffness-to-weight ratios, and are used in different industries such as aeronautics and aerospace, F-1 cars, sport accessories, and ship construction. However, traditional manufacturing processes for composites require qualified operators and/or expensive equipment for developing time-consuming manufacturing operations, resulting in high costs. Additionally, the fabrication of parts with complex geometries is difficult. These manufacturing drawbacks can be mitigated by using composite 3D printing technology. In fact, 3D printing is becoming a potentially attractive manufacturing technique in industrial applications due to design flexibility, reduced assembly time, low volume production, low amount of waste material, improved recyclability, etc. Potential applications for the 3D printing technology include the aerospace industry, architectural industries for structural models, printing tissues, organs and art [1,2,3,4,5].

In the frame of additive manufacturing of composite materials, the most suitable technology is Fused Deposition Modeling (FDM) or Fused Filament Fabrication (FFF). This is because of its relative low cost, low material waste, high accuracy and wide range of machines, from industrial to home-made printers. On the other hand, this kind of technique can induce several internal defects during the manufacturing process due to the lack of a compaction stage between layers. These internal voids can greatly reduce the stiffness and strength of a final printed part. These flaw zones can act as stress concentrators and parts can fail prematurely. The use of fibers to reinforce the material not only mitigates the loss of properties due to voids but improves its mechanical performance. The effect of using short fiber reinforcements on the mechanical properties of the material in FDM has been extensively analyzed. Most studies have focused on the in-plane longitudinal tensile test [6,7,8,9,10,11,12] and in-plane transverse tensile test [6,7,9,11,12]. The most commonly used composite are Acrylonitrile Butadiene Styrene (ABS) and short carbon fiber (SCF) at a different weight percent (wt.%). Only marginal improvements of the mechanical properties with respect to neat polymers, especially in terms of strength, have been reported. Different authors have also tried to improve the mechanical properties of 3D-printed parts, namely stiffness and strength, by optimizing the process parameters, such as the infill fiber pattern participation, build orientation and thickness layer [13,14,15,16]. However, the resulting mechanical properties are still low if compared to traditional manufacturing methods such as injection molding, compression molding or vacuum bags [17]. This explains why 3D printing of continuous fiber reinforced composites arises as a promising technology with improved mechanical properties with respect to any discontinuous reinforcement system [18,19,20].

The number of published studies on the mechanical properties of 3D-printed continuous fiber-reinforced composites is limited in comparison to the case of materials with discontinuous reinforcement. One of the reasons for this is that to date there are only two commercial FDM 3D printers capable of working continuous fiber-reinforced polymer [4,13,14,20,21,22,23]. MarkForged MarkTwo^®^ [24] and Anisoprint Composer^®^ [25] can build up parts with continuous and aligned fiber-reinforced composite materials. The first is based on the extrusion of a fiber filament pre-impregnated with polyamide and can use carbon, glass and Kevlar^®^ fiber to reinforce neat polyamide or polyamide already reinforced with short carbon fiber. The Anisoprint Composer^®^ system can use either continuous carbon or basalt fiber to reinforce any of the usual thermo-plastic polymer filaments used in FDM. In this case, the reinforcement process is based on the co-extrusion of the thermoplastic material with the fiber filament already impregnated with a thermoset resin.

In most cases the mechanical properties of the printed material are assessed using the in-plane tensile test [13,14,16,18,19,21,26,27,28,29,30]. This test has also been used for the specific case of the carbon fiber composite used in the MarkForged MarkTwo^®^ 3D printer [27,29,31,32,33,34,35] with high variability in the results (see Section 4). Some authors used the ASTM D638 [36] standard or a variation of this, such as the one proposed by Croccolo et al. [37], while others used the ASTM D 3039 [38]. In their work, Pyl et al. [29] established a comparison between these three different specimen geometries and concluded that the best results were achieved by using the ASTM D3039 specimen with paper end-tabs, while all the ASTM D638 samples prematurely failed at the starting location of printed reinforcement [13,14,29]. Flexural tests have been also used to assess the material properties [14,16,18,28]. Chacon et al. [22] and Iragi et al. [27] used the Interlaminar Shear Strength test to characterize the material. Goh et al. [28] used the quasi-static indentation test to determine the material flexural behavior. The impact damage resistance of the material was investigated by Caminero et al. [23] using the Charpy pendulum. The same authors evaluated the internal material damage after a low-velocity impact event using ultra-sonic inspection [39].

Although the flexural behavior of the material is highly affected by its intralaminar and interlaminar fracture toughness, there are few works devoted to the subject in the literature. Focusing on the interlaminar behavior, only few studies on mode I fracture toughness of 3D-printed composite materials are available. The work of Aliheidari et al. [40] is one of the first to characterize the fracture toughness in mode I of a 3D-printed sample of neat ABS. They found that the higher the extrusion temperature, the higher the fracture resistance, with maximum reported values close to the fracture resistance of bulk ABS. Young et al. [17] studied the fracture toughness of both neat ABS and SCF/ABS 15% wt using the DCB specimen (ASTM D5528 [41]). To create the precrack during the manufacturing process, a designed printed tooling was used for placing a Kapton^®^ tape at midplane of the FFF specimen without stopping the printing process. Glass/epoxy doublers were bonded to the DCB printed specimen to reach the usual thickness and avoid arms’ failure during loading. While the neat ABS specimens showed stable crack propagation, a combination of stick-slip behavior and stable growth was found for the reinforced specimens. The authors also found that the fracture toughness of the FFF samples was lower than that of hot-press molded specimens and the FFF specimens reinforced with short carbon fiber presented lower fracture toughness than neat ABS. Khudiakova et al. [42] experimentally determined the stress intensity factor and the fracture toughness in mode I of FFF net PLA and SCF/PLA specimens with different thickness using both the DCB test and Cracked Round Bar. The fracture toughness of the net polymer was found to be almost twice that of the SCF/PLA. During the test of hybrid specimen the fracture propagation took place in the reinforced material part due to its lower fracture toughness. Fonseca et al. [43] analyzed the interlaminar fracture toughness of 3D-printed specimens of neat PolyAmide-12 (PA12) and SCF/PA12 15% wt without using any reinforcement. In fact, they considered that using doublers may increase the overall fracture toughness because damage can develop at the interface between the specimen and the doublers, increasing the fracture energy dissipated. A Kapton^®^ film was placed in the midplane during printing process using a designed tool for creating precrack. During testing unstable crack growth was observed for all samples. In contrast to the works of Young et al. and Khudiakova et al., reinforced systems revealed a strong increase of material toughness in relation to non-reinforced systems.

The work of Iragi et al. [27] experimentally evaluates the interlaminar fracture toughness of the 3D-printed continuous carbon fiber-reinforced PA composite used in the Markforged MarkTwo^®^ 3D printer (CF/PA). The authors analyzed this fracture toughness both in mode I (*G*_I_) and mode II (*G*_II_) following the DCB [33] and ENF [44] tests, respectively. To prevent the failure of the arms during the tests, the same approach used by Young et al. [17] was used and 3 mm thick Dyneema^®^ doublers were bonded to the CF/PA specimens. Thus, the total thickness of the specimens was 9 mm. It was found that, contrary to the common tendency in composite materials, the initiation fracture toughness in mode II (*G*_IIc_ = 1.59 kJ/m^2^) was lower than that in mode I (*G*_Ic_ = 2 kJ/m^2^). The authors justified this unusual material behavior by the large number of fiber bridges in mode I tests and the lack of matrix shearing in mode II tests. However, the use of doublers may have affected the results due to the presence of additional damage in the composite/doubler interfaces, as considered by Fonseca et al. [43]. Additionally, the use of such thick specimens, almost twice the limit indicated in the standard (5 mm), may also have affected this measure. In fact, the thickness of the specimen has been observed to have an effect on the measure of the interlaminar fracture toughness in composites. Farmand-Ashtiani et al. [45] analyzed the effect of the thickness of DCB in composite specimens and found that there is no effect on the initiation value of *G*_Ic_ but the propagation value increases with the thickness of the specimen. On the other hand, Kumar et al. [46] observed that the initiation value decreases for thicker specimens and no clear tendency was found for the propagation value. To the best knowledge of the authors, there is no other reference for the fracture toughness values of this material. Thus, and taking into account that the reported values by Iragi et al. may have overestimated *G*_Ic_, additional experimentation and further analysis is necessary to increase knowledge about the facture behavior of this material.

This work focuses on the experimental characterization of the initiation and propagation interlaminar fracture toughness in mode I and mode II of Markforged MarkTwo^®^ continuous carbon fiber-reinforced PA composite without using composite doublers. The effect of the specimen thickness on the mode I fracture toughness is also investigated by considering two different specimen thicknesses. On the other hand, the in-plane mechanical properties of the material reported in the literature show high variability (see Section 4) and the elastic properties of 3D-printed composites are affected by the processing parameters of the 3D-printer, as well as the specimen geometry or use of end-tabs. Thus, the in-plane mechanical properties of the material (longitudinal tensile, transverse tensile and shear) have also been determined using different test standards and end-tabs to characterize the material and establish a comparison. After the analysis it can be concluded that the use of composite doublers and too-thick delamination specimens may affect the interlaminar fracture toughness characterization of the 3D-printed CF/PA composite used in the Markforged MarkTwo^®^ 3D-printer. Furthermore, the geometry of the specimen affects the characterization of the in-plane mechanical properties with premature failures in some cases.

## 2. Materials and Methods

The material considered in this study is the 3D-printed composite material obtained with the Markforged MarkTwo^®^ (Watertown, MA, USA) filament combining continuous carbon fiber with PolyAmide, CF/PA (which is the only material combination for a carbon composite in this 3D-printer model). As commented before, the characterization of the interlaminar fracture toughness depends on the elastic properties of the material, which in the case of the 3D-printed composite are highly dependent on the printer configuration. Thus, this section initially describes the procedures to determine the in-plane elastic properties of the composite material and the printing configurations. The procedure for manufacturing and testing the interlaminar fracture toughness of mode I and mode II specimens without using composite doublers is also described in detail. Two different specimen thicknesses are taken into account to evaluate the thickness effect on the crack propagation in mode I.

The reinforcing filament used in the Markforged MarkTwo^®^ 3D-printer contains a thousand carbon fibers (with Young’s modulus *E* = 54 GPa and tensile strength *X*^t^ = 700 MPa) embedded in a PA matrix (*E* = 0.94 MPa, *X*^yield^ = 34 MPa and *X*^t^ = 54 MPa). The cross-section of the CF/PA filament was measured using microscopy and image analysis and it was found that the fiber volume fraction is 35% with many resin rich areas and voids. Other authors have reported fiber volume fraction values between 27 and 41% and also many resin rich and dry spot areas [16,28,30,33]. The nominal thickness of the CF/PA plies is 0.125 mm (determined by the 3D printer) with solid infill pattern. It is worth noting that nozzle diameters and temperatures or printing velocities are determined by the manufacturer and cannot be modified during the printing process. In addition, the manufacturing procedure of the printing system imposes that all the carbon-fiber reinforced layers must be internal and covered with nylon (PA-6). Therefore, the CF/PA layers are always sandwiched between nylon layers oriented at 45 or −45°. The thickness of the nylon layers is also 0.125 mm, and a solid infill was used. The number of nylon outer walls was set to be equal to the number of nylon roof and floor layers for all specimens.

The use of a high amount of carbon-fiber reinforcement, as required in the characterization specimens of the CF/PA composite, causes adhesion problems between the part being manufactured and the printer bed, complicating or even invalidating the manufacturing process. Instead of manufacturing composite plates from where the individual specimens could be cut, all the specimens were manufactured individually (2 or 3 specimens can be manufactured at the same time) and with nylon skirt and rounded corners to avoid debonding between part and printer bed.

### 2.1. Manufacturing and Testing of the In-Plane Tensile and Shear Characterization Specimens

Initially the specimens for the characterization of the in-plane tensile properties were manufactured according to the ASTM D638 standard, common for the characterization of polymer materials, and avoid the use of end-tabs. For the longitudinal direction the specimens were printed with all the fiber beads aligned in the longitudinal direction of the specimen, 0°. For the transverse direction, the specimens were printed with all the fiber beads perpendicular to the longitudinal direction of the specimen, 90°. The in-plane shear specimens were manufactured alternating layers with all the fiber beads at +45 and layers with all fibers at −45°. After reviewing the results, the in-plane transverse characterization was deemed to be correct. However, it was found that for the longitudinal and in-plane shear tests the mechanical properties obtained were lower than expected and most of the failures were located in the transition zone, out of the gauge length. Moreover, the in-plane shear specimens failed before reaching 5% of shear strain. A second batch of specimens for longitudinal tensile and in-plane shear properties was manufactured based on the ASTM D3039 standard for composites with the use of paper end-tabs as suggested by Pyl et al. [29]. So, an additional comparison between the results obtained with the two different specimen geometries and the use of end-tabs can be performed. Table 1 summarizes the geometries/standards considered for the tests as well as the lay-up of the different specimens in each case.

The nylon cover of the specimens was not removed after the manufacturing process to simplify their post-processing and for being more representative of a possible printed part. Although the influence of the nylon cover is minor with respect to the carbon fiber reinforced layers (especially in the case of the longitudinal specimens), it was not taken into account during the analysis of the experimental data and only the cross-section of the CF/PA material was considered. To prevent the moisture absorption of the nylon and avoid any possible influence on the experimental results [47,48,49], after manufacturing all the printed specimens were stored inside a dry-box with several desiccant bags at room temperature until they were instrumented and tested.

Five specimens were manufactured and tested for each one of the combinations. All the in-plane tests were carried out at 23 ± 2 °C and 50 ± 5% HR using an MTS Insight testing machine equipped with a 50 kN load cell at a displacement rate of 2 mm/min. During the tests, the load and displacement signals of the testing machine and longitudinal and transverse strain in the specimen were recorded.

### 2.2. Manufacturing and Testing of the Interlaminar Fracture Toughness Specimens

**Figure 1:**
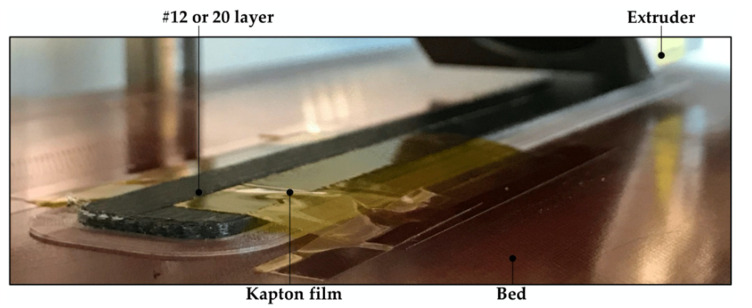
Placement of the adhesive Kapton^®^ tape at midlayer of the DCB specimen: after layer 12 or 20 for the DCB-A and DCB-B specimens, respectively.

**Figure 2:**
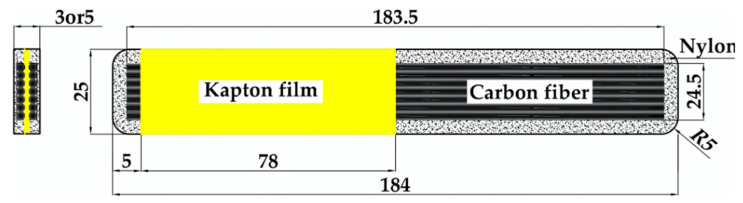
Schematic top view and cross-section of the DCB specimens for mode I fracture toughness characterization with the corresponding dimensions.

The DCB specimens for the characterization of the mode I fracture toughness were printed according to the ISO-15024 standard [50]. Two different thicknesses were considered for the specimens’ arms to capture the effect of this thickness in the measure of the fracture toughness. The thickness of the arm for the thin specimens, DCB-A, was set to 1.5 mm (3 mm in total), while for the thick specimen, DCB-B, it was set to 2.5 mm (5 mm in total). To generate the starter crack at the specimen’s midplane, once the bottom half was fully printed (after layer 12 and layer 20 for the DCB-A and DCB-B specimens, respectively), the printing process was paused. Then, a film/tape was laid on top of the appropriate area and fixed to the printer bed. In a first attempt the typical PTFE film used in composite materials was used. However, the results were not correct because the heat of the printer head generated several wrinkles in the PTFE film resulting in imperfections in the top part of the specimen (waviness and fiber misalignment) and obstructions in the printer head. In a second attempt, adapting the procedure used by Fonseca et al. [43] an adhesive Kapton^®^ tape and a holding support were used but with unsatisfactory results. The adhesion between the Kapton^®^ tape and the deposited CF/PA filament resulted to be unsatisfactory (the adhesion with pure PA filament was correct). Besides, it was not possible to achieve enough tension in the tape using the holding support, which resulted in waviness and fiber misalignment. Hence, the adhesive Kapton^®^ tape was laid on top of the bottom half of the specimen and fixed to the printer bed face-up (adhesive part on top) with an auxiliar tape. An additional area without film beyond the pre-crack area was left to ensure good adhesion. It was possible to achieve the necessary tension in the tape and avoid wrinkles and fiber waviness (see Figure 1 and Figure 2) while the printed carbon fiber filament bonded to the tape. Obviously, the additional area without film had to be removed/cut before testing, as it would prevent the propagation of the crack.

Taking into account the relatively low strength and high fracture toughness reported by Iragi et al. [27] for the same material and the lack of experience in the characterization of the interlaminar fracture toughness without using doublers or reinforcements, the geometry of the specimens was modified to avoid premature failure. A preliminary batch of 200 mm long DCB-A and DCB-B specimens with 100 mm long pre-cracks was prepared to ensure the propagation of the interlaminar crack avoiding excessive bending and failure in the arms. Although the geometry of these specimens was not according to the standard, these preliminary tests served to assess that it was possible to obtain crack propagation before the failure of the arms and that the standard geometry could be used. Thus, a new batch of DCB-A and DCB-B specimens was manufactured with the standard length (Figure 2 and Table 2). It is worth mentioning that the afterwards comparison of the results showed that the value of the measured mode I fracture toughness was similar for the standard and the preliminary long DCB specimens.

As shown in Figure 2, pure nylon areas (dotted zones) covered the continuous carbon fiber aligned at 0° (black areas). The Kapton^®^ adhesive tape was placed on top of both the pure nylon and carbon reinforced section to generate the starter crack. All the nylon zones and the last 5 mm of CF/PA covered with Kapton^®^ were cut using a diamond saw before the tests.

Regarding the characterization of the interlaminar fracture toughness in mode II, the End-Notched Flexure (ENF) test (ASTM D7905 [44]) was used to determine the initiation value. The Calibrated End-Loaded Split (C-ELS) test (ISO 15114 [51]) was used to obtain both the initiation and propagation values. In both cases, the test specimens were manufactured and post-processed following the procedures described for the DCB specimens. Table 2 summarizes the characteristics of the mode I and mode II specimens manufactured. After the analysis of the results in mode I (Section 3 and Section 4), it was decided to only consider 3 mm thick specimens for the characterization of the mode II interlaminar fracture toughness of the material

**Figure 3:**
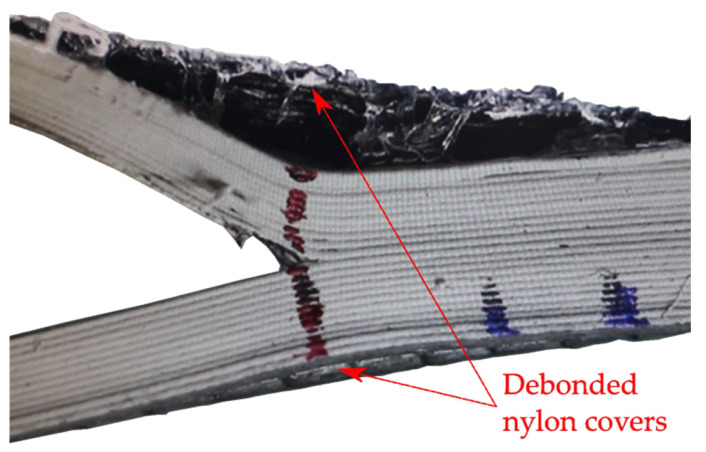
Close-up of the crack tip area of a DCB-A specimen showing the debonding of the nylon covers from the rest of the specimen.

To avoid any interference in following the progression of the crack during the test, the additional nylon in the laterals of the specimen was cut using a diamond saw but the top and bottom nylon surfaces were not removed. However, as it can be seen in Figure 3, these nylon covers tended to debond from the rest of the specimen and deform in a different way, invalidating the test. To overcome this issue the printing process was stopped just before the top nylon surface was printed but as the bottom nylon cover is inherent of the printer process, it cannot be avoided. Thus, it was also necessary to cut the nylon at the bottom with the same diamond saw before the test. The final dimensions of the interlaminar specimens after the post-processing are summarized in Table 2.

To facilitate tracking the crack propagation during the test, the edges of the specimens were painted with an appropriate white paint and the propagation lines indicated in the standard were marked. Part of the top and bottom surfaces of the DCB specimens and bottom surface of the C-ELS specimens were abraded using an 80-grade sandpaper creating a longitudinal and transversal texture. Aluminum MW2001-6 loading blocks were adhered to these specimens by coating surfaces with Henkel-Ibérica Loctite^®^ 401 (Barcelona, Spain). The adhesive was let to cure at room temperature for 24 h using hand-presses to fix sample and loading blocks properly. One NA3-30 inclinometer with a resolution of 0.005° was attached to each loading block of the DCB specimens to track their inclination during the test and be able to determine the interlaminar fracture toughness in mode I using the J-integral method according to Paris and Paris [52] and Sarrado et al. [53]. Both the initiation, *G*_Ic,ini_, and propagation, *G*_Ic,prop_, values were considered. For the C-ELS specimens, also the initiation and propagation values, *G*_Iic,ini_ and *G*_Iic,prop_, were calculated by means of the Corrected Beam Theory with effective crack length (CBTE), as indicated in the standard [51]. For the ENF test, the initiation mode II fracture toughness was determined according to the Compliance Calibration (CC) reduction method, also as indicated in the standard [44].

**Figure 4:**
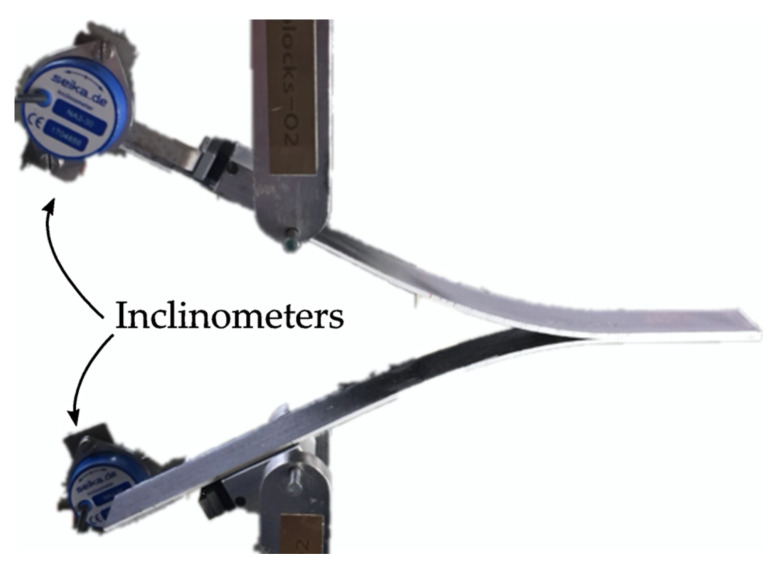
DCB-A specimen with loading blocks and inclinometers during a DCB test.

All the interlaminar fracture tests were carried out using an MTS (Eden Prairie, Minnesota, MN, USA) Insight testing machine equipped with a 5 kN load cell at 23 ± 2 °C and 50 ± 5% HR. The cross head speed was set to be 5, 1 and 1 mm/min for the DCB, ENF and C-ELS tests, respectively, for loading and 25, 10 and 10 mm/min for unloading. In all the cases, before the real test, the initial pre-crack from the Kapton^®^ adhesive tape was extended between 3 and 5 mm in mode I, using the DCB tests to avoid any possible effect of the tape and ensure the initiation from a sharp crack tip. During all tests, force and displacement signals were recorded, as well as the signal from the inclinometers in the DCB tests. The crack propagation was tracked and recorded using a high-resolution video camera for the both DCB and C-ELS specimens. The crack propagation was not monitored for the ENF tests because it is typical for this type of test that the propagation is unstable, growing instantaneously from the pre-crack to bellow the load roller, where it is arrested. Figure 4 shows a DCB-A specimen during the test with the loading blocks and the inclinometers. As it can be seen in the image, the opening of the arms is totally symmetric and with the typical deformed shape under bending.

**Figure 5:**
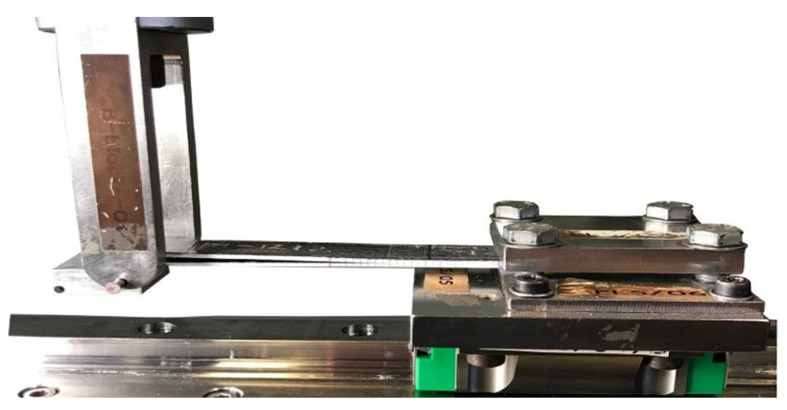
C-ELS specimen mounted on the C-ELS test rig for the mode II test.

Prior to perform the C-ELS tests, a clamp calibration was performed at 50, 60 and 70 mm length, as indicated in the standard. Hence, the specimen flexural modulus (*E*_f_) and the C-ELS clamp correction were obtained to use the CBTE reduction method [51]. During the test, the un-cracked end of the sample was fixed to the sliding clamping system of the test apparatus so it could move horizontally when a vertical displacement was applied to the loading block on the bottom surface of the specimen at the cracked end, as shown in Figure 5.

Five DCB-A and three DCB-B specimens were manufactured, prepared and tested to determine the mode I interlaminar fracture toughness. Three ENF specimens were used to determine the initiation value of *G*_Iic_, and five C-ELS specimens were considered for both the initiation and propagation values of *G*_Iic_. After, the specimens were manually split open for fracture surface inspection and representative samples inside of the crack propagation area were prepared for the fractographical analysis using a Zeiss (Oberkochen, Germany) DSM 960A SEM at several magnification factors.

## 3. Results

The results of the characterization of the in-plane tensile and in-plane shear material properties and the interlaminar fracture toughness are presented next. It is worth noting that in-plane tensile and in-plane shear results only take into account the CF/PA cross-section of the material. Thus, the nylon walls and top and bottom covers are not taken into account even if they were not removed from the specimens before the tests.

### 3.1. In-Plane Material Properties

**Figure 6:**
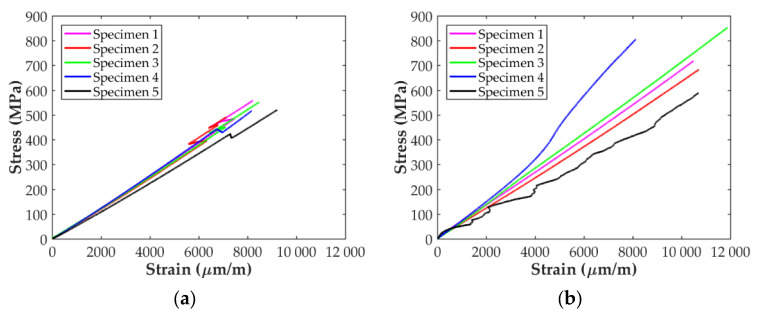
Longitudinal stress-strain curves of the (**a**) ASTM D638 specimens and (**b**) ASTM D3039 specimens with paper end-tabs.

The stress-strain curves of the longitudinal tensile tests obtained with the D638 and D3039 specimens are shown in Figure 6. For the D638 specimens, Figure 6a shows that the stress state increases linearly with the applied strain up to around 6000 μm/mm. At this strain level a sudden strain change, probably due to a stress concentration in the area of the fillet, can be observed for all the specimens. After this point, the stress level in the specimens continues increasing with the strain until the final abrupt failure at maximum tensile stress, as expected for this type of material. The stress-strain curves for the longitudinal ASTM D3039 specimens using paper end-tabs are presented in Figure 6b. Although in two cases the corresponding curves show a strange behavior (probably due to an error in the extensometer), the stress level increases linearly with the applied strain until a final brittle failure occurs, as for the D638 specimens. Indeed, the stress curve for Specimen 4 experienced a smooth increment at around 4500 μm/mm, as if a stiffening process was present in the specimen. With respect to Specimen 5, the entire experimental data exposed a peculiar performance, which can be attributed to a problem with the strain transducer. Therefore, these two specimens were not taken into account during the data reduction process to determine the longitudinal in-plane properties of the material with the D3039 specimens.

**Figure 7:**
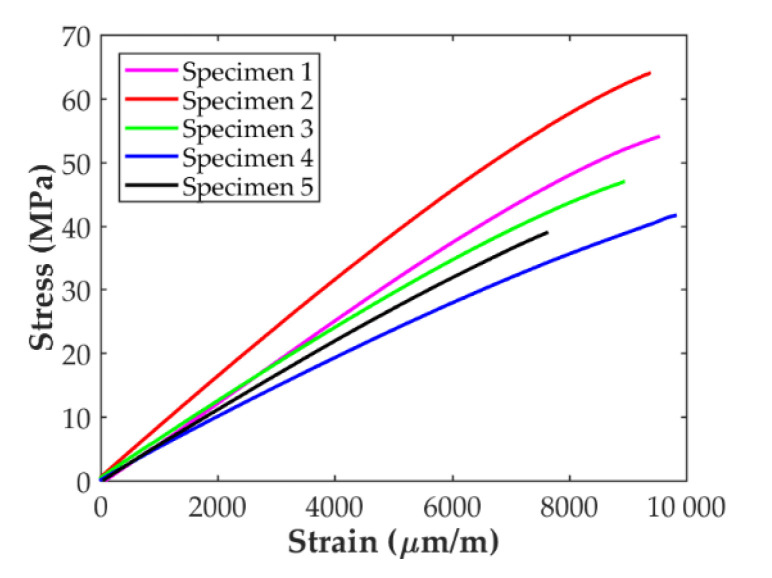
Stress-strain curves for the transverse specimens.

The stress-strain curves for the five D638 specimens tested in the transverse direction are presented in Figure 7. As for the longitudinal specimens, the stress increases almost linearly with the applied strain for all the samples until brittle failure occurs. All the curves follow the same trend except for one, Specimen 2, in which the slope of the linear part of the curve and the level of maximum stress is higher than for the rest.

**Figure 8:**
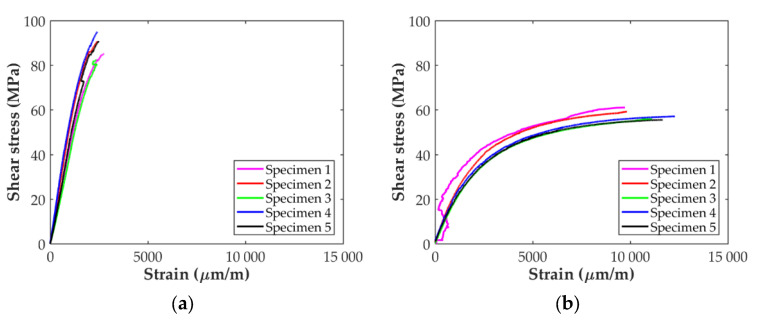
In-plane shear strain-stress curves: (**a**) ASTM D638 and (**b**) ASTM D3039 specimens.

The resulting stress-strain curves for the in-plane shear tests are shown in Figure 8. In all cases, the curves show a linear behavior at the beginning until the response starts to become clearly non-linear. In the case of the ASTM D638 specimens, Figure 8a, the non-linear response is accompanied with at least one sudden strain variation in the range from 1500 to 2500 μm/mm, probably due to a stress concentration in the area of the fillet, as in the case of the longitudinal specimens. The strain-stress curves for the D3039 in-plane shear specimens are shown in Figure 8b. It can be observed that the curve for one of the specimens shows an unexpected behavior. This is attributed to an error of the strain sensors used during the test and this specimen was not considered for the determination of the in-plane shear properties. For the rest of the specimens the linear response is observed until a strain level of about 2%.

The results of the in-plane tensile tests (longitudinal and transverse directions and in-plane shear) are summarized in Table 3.

### 3.2. Interlaminar Fracture Toughness

**Figure 9:**
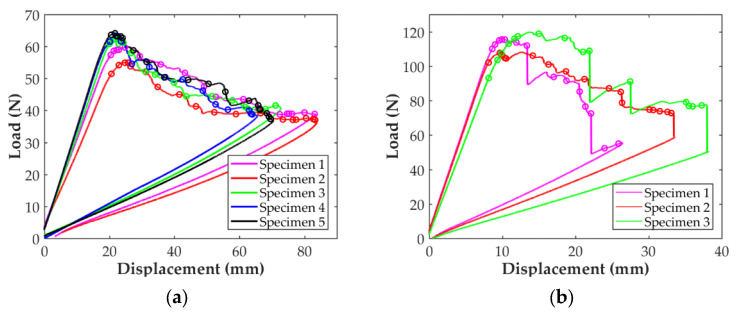
Load-displacement curves for the (**a**) DCB-A specimens and (**b**) DCB-B specimens. Marks correspond to the different crack propagations indicated in the standard.

The load-displacement curves for the DCB tests can be seen in Figure 9. The marks in the curves correspond to the different crack propagations indicated in the standard. It can be observed in Figure 9a that the trend is very similar for all the DCB-A specimens, with smooth and stable crack propagation. On the other hand, the load-displacement curves for the DCB-B specimens, Figure 9b, show the typical load drops associated to a stick-slip behavior with rapid crack propagations combined with crack arrests. Besides, the load-displacement curves for the three DCB-B specimens show certain differences, especially Specimen 1 DCB-B, for which the maximum displacement achieved was clearly lower even if the final crack propagation was similar to the other two specimens. This resulted in a smaller area bellow the load-displacement curve for this specimen and a value of the calculated interlaminar fracture toughness about 25% lower. Thus, it was decided to exclude this specimen from the determination of *G*_Ic_.

**Figure 10:**
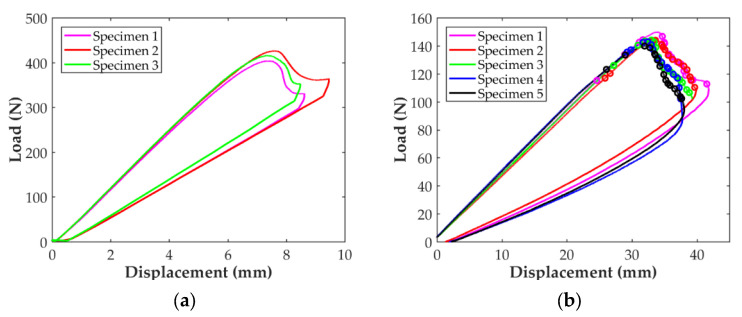
Load-displacement curves for the (**a**) ENF specimens and (**b**) C-ELS specimens. Marks correspond to the different crack propagations indicated in the standard.

The mode II load-deflection curves of the three ENF specimens and the five C-ELS specimens are shown in Figure 10. All curves for each specimen type exhibit the same trend with good repetitiveness: load increases linearly with the applied displacement almost up to the peak load. After this point, the load tends to decrease in the typical non-linear trend, until the test was stopped for the ENF specimens, Figure 10a, and until the end of the propagation marks for the C-ELS ones, Figure 10b. As it normally occurs for the ENF test, the crack propagated in a rapid and unstable way until it was located under the load roller and the test was stopped. For this reason, only the initiation value of *G*_II_ was determined for the ENF test. Crack propagation was continuous and smooth for C-ELS specimens and both initiation and propagation values were determined.

The results for the interlaminar fracture toughness tests of the CF/PA composite are summarized in Table 4.

## 4. Discussion

### 4.1. In-Plane Material Properties

**Figure 11:**
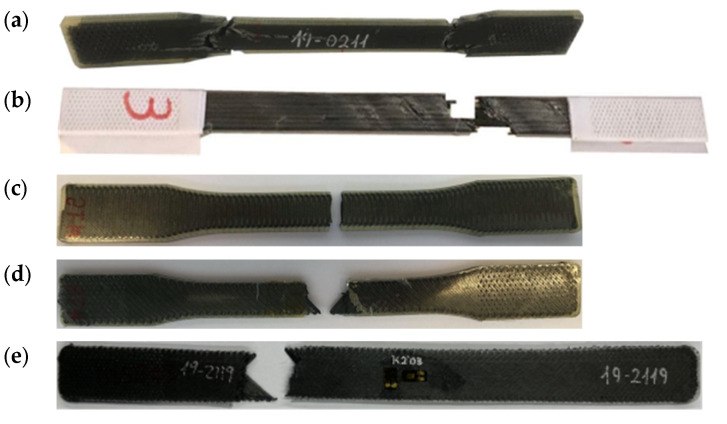
Representative failure type for each in-plane specimen configuration: (**a**) longitudinal ASTM D638 with failure at both fillet areas, (**b**) longitudinal ASTM D3039 with paper end-tabs with lateral failure in the gauge length, (**c**) transverse ASTM D638 with lateral failure, (**d**) in-plane shear ASTM D638 with typical ±45° failure and (**e**) in-plane shear ASTM D3039 with typical ±45° failure.

Comparing the results shown in Figure 6 and Table 3, it can be seen that the value of the longitudinal modulus and the strength are higher for the D3039 specimen, 16% and 42% higher, respectively. Thus, it can be considered that, in terms of the characterization of the elastic behavior, both specimens are similar. However, the stress concentration generated in the fillet of the D638 specimens combined with the fact that the 3D printer starts and ends the deposition of the carbon filament in this area results the premature failure of the specimen. Similarly, the stress loss shown in Figure 6a for all the D638 specimens before the final failure (between 6000 and 7000 μm/mm of axial strain) can be attributed to the same. This explains why the failure for all the D638 specimens was located at the fillet area and outside the gauge length, as shown in Figure 11a. A similar observation was previously reported in the literature [5,14,15,29]. On the contrary, the prismatic geometry of the D3039 specimens with paper end-tabs, as suggested by Pyl et al. [29], improves the load transfer between the testing machine and the specimen, reducing the stress concentration in the clamping area. This is why the level of maximum stress is higher for all the D3039 specimens and all failures were located at the gauge length with lateral fracture, as shown in Figure 11b. Consequently, the two test configurations are equivalent for the determination of the axial modulus in the longitudinal direction but for the determination of the tensile strength and ultimate strain it is better to use ASTM D3039 specimens with paper end-tabs.

**Figure 12:**
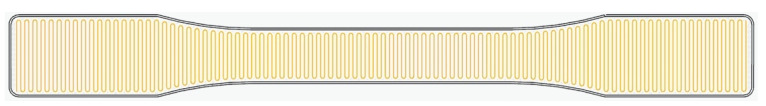
Schema of the transverse tensile specimen as obtained in Eiger (Markforged’s slicer program) with the trajectory turns at the edges of the specimen. Orange line indicates the trajectory of the fiber filament and dark line indicates the trajectory of the nylon wall.

Analyzing the results for the transverse tensile specimens, it is seen that the resulting stress-strain curves are similar for all specimens but for one, which shows a stiffer and higher strength value. Contrary to the case of the D638 longitudinal tensile specimens, the response of the transverse tensile ones has not been affected by the fillet. Indeed, all failures for these specimens took place in the gauge area, as shown in Figure 11c. Taking into account that in this case the carbon fiber reinforcement is laid perpendicular to the load direction, its contribution should be small and the resulting material properties should be close to those of the pure nylon. Comparing the results of the transverse specimens (summarized in Table 3) with those reported by Markforged [24] for the nylon (Section 2), it is observed that the 3D-printed CF/PA is 6.55 times stiffer and has a similar strength. However, it is more fragile: about 1% elongation vs. 260%. This difference in stiffness is attributed to the fact that these specimens were manufactured in their final shape and without any post-process cutting. Thus, the carbon fiber beads were deposited transversally to the longitudinal direction of the specimens except at the edges, where a U-turn is necessary, as shown in the schema from Eiger (the slicer for the Markforged MarkTwo), Figure 12. This means that part of the fiber filaments, albeit small, worked parallel to the applied load during the test, resulting in a higher transverse Young’s modulus. A similar observation was reported by Todoroki et al. [34], who tested transverse specimens with and without fiber U-turns (serpentine folded fibers) at the edges and found that the transverse Young’s modulus is higher in the first case.

The results for the in-plane shear tests presented in Figure 8 indicate that the material response with the D638 specimens is stiffer and reaches higher values of shear stress than for the D3039 specimens. However, while the stress-strain curves in Figure 8b show a plastic/ductile response for specimens D3039, as it was expected, the material behavior shown in Figure 8a for the D638 specimens is brittle. Taking into account the in-plane shear results summarized in Table 3, the in-plane shear modulus determined with the D638 specimen is about 2.3 times higher than for specimen D3039. Similarly, the maximum shear stress for specimen D638 is about 56% higher than for the D3039. On the contrary, the maximum shear strain for the D3039 case is about five times higher than for the D638 specimens. In fact, the level of 5% in shear strain indicated in the ASTM D3518 standard to determine the in-plane shear strength of the material was not reached with the D638 specimens (only 2.5% was achieved). All these observations indicate that the geometry of the D638 specimens imposes restrictions to the free deformation of the material under in-plane shear loading while this is not the case for the D3039 specimens. On the one hand, the lower width of the D638 specimen (13 mm versus 25 mm in D3039 specimens) generates an edge effect that limits the alignment of the fiber filaments with the load direction. On the other hand, the larger width of the specimen in the clamping area also limits the rotation of more fiber filaments, whereas the combination of a longer geometry and the use of paper end-tabs (D3039 case) does not impose such a restriction in the fibers rotation. As shown in Figure 11d,e, all the specimens exhibited the typical ±45° shear failure located at the gauge length. In consequence, for the determination of the in-plane shear material properties it is better to use specimen D3039.

Comparing the results in Table 3 and in Figure 6, Figure 7 and Figure 8 (only the specimens taken into account), it is observed that the variability is higher for the in-plane transverse direction. Finally, the in-plane tensile and shear mechanical properties of the CF/PA composite obtained in this work can be compared with those available in the literature and summarized in Table 5. The dispersion in the values is clear. For instance, the transverse Young’s modulus reported by Yogeshvaran et al. [35] is 8 times higher than the value reported by Pyl et al. [29]. This dispersion in the results, especially for transverse and in-plane shear, can be partly explained by the fact that during the manufacturing process multitude of voids are generated within the material, especially between the deposited filaments, as indicated by different authors [27,29,31,32,33,34,35], affecting the behavior of the material in the transverse direction. As it is well known, the settings of each individual 3D printer affect the compaction level, which influences the amount of porosity and the final mechanical properties of the printed parts. In the transverse direction, another cause of variability in the results is related to the orientation of the fiber beads at the edges of the specimens. Iragi et al. [27] manufactured larger plates from where the individual specimens were cut, ensuring that along the width of the specimen the fiber filaments were oriented transversally to the applied load. Todoroki et al. [34] used specimens with and without U-turns at the edges of the specimens showing that the Young’s modulus and transverse strength can be 25% higher if U-turns are present. No information was provided on this by Yogeshvaran et al. In the case of Pyl et al. they only tested one transverse specimen using the D3039 configuration. The authors reported a transverse Young’s modulus of 1.46 Gpa but taking into account the whole specimen’s cross-section (nylon included) and did not report the strength value. Analyzing the data included in their work, it can be inferred that the Young’s modulus of the CF/PA material for this single specimens is 1.83 Gpa and its strength is about 40 Mpa. Comparing the results obtained in this work with those previously reported in the literature, it is observed that all the values for all the material properties determined fall within the range of values in the literature. Only the value of the transverse strength is out of the range, which is deemed to be caused by the U-turns of fiber at the edges of the specimen.

### 4.2. Interlaminar Fracture Toughness Characterization

**Figure 13:**
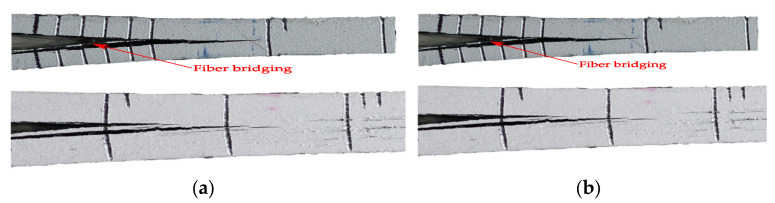
Close-ups of the crack tip areas during tests with details of the delamination extension: (**a**) DCB-A specimen with fiber-bridging and crack branching and (**b**) DCB-B specimen with crack branching and jumping and propagation of multiple cracks.

The results for the interlaminar fracture toughness in mode I reported in Figure 9 and Table 4 show that the thickness of the specimen clearly affects this characterization. On the one hand, a stick-slip behavior is detected in the propagation of the crack for specimens DCB-B, while the propagation in the thinner specimens is smooth. On the other hand, both the initiation and propagation values of *G*_I_ are affected by the thickness of the specimen. The values of *G*_I,ini_ and *G*_I,prop_ are 41and 36% higher for specimen DCB-A, respectively. In fact, during the tests few sporadic fiber-bridging events were detected for specimens DCB-A, although some crack branching was also observed, as shown in Figure 13a. In the case of the DCB-B specimens, fiber-bridging, crack branching and crack jumping were common, as shown in Figure 13b, compromising the validity of the test results. Another disadvantage found with the thicker DCB specimens was that after the tests, a permanent plastic curvature of the specimens’ arms was observed, while for the thinner specimens this was not observed. Therefore, it can be concluded that for the determination of the mode I interlaminar fracture toughness of the CF/PA material, it is better to avoid the use of thick specimens. This is the reason why it was decided to only continue processing the results for specimens DCB-A and only consider 3 mm thick specimens for the characterization of the mode II interlaminar fracture toughness of the material.

**Figure 14:**
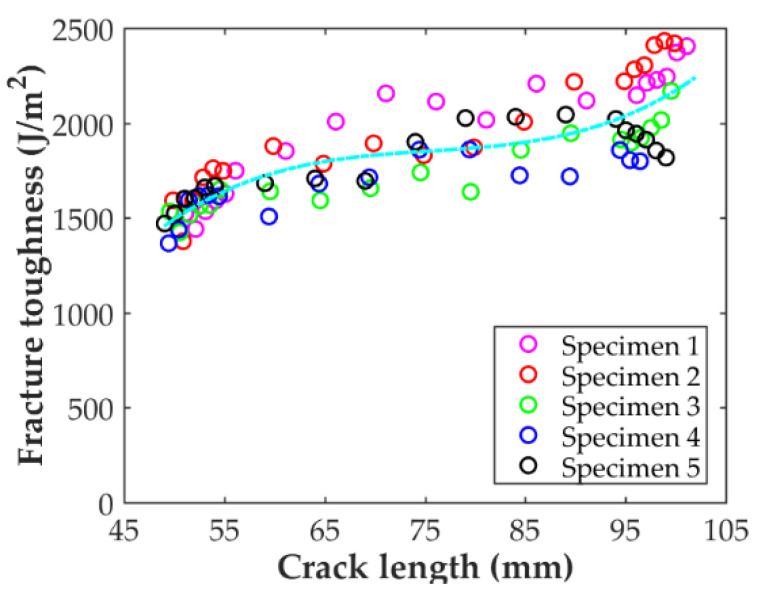
Crack propagation resistance curve for the DCB-A specimens.

The crack propagation resistance curve, R-curve, for the DCB-A samples is shown in the Figure 14. It is seen that the curve tends to increase for short and long crack lengths but remains constant for the central range of values. For crack lengths between 60 and 90 mm, the fracture toughness value is fairly constant and in good agreement with the average propagation value reported in Table 4, 1720 J/m^2^. A similar tendency for the R-curve was reported by Iragi et al. [27] for the same material.

**Figure 15:**
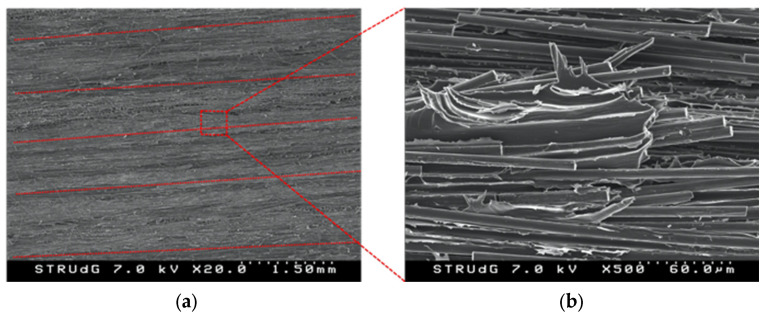
SEM images of the fracture surface of a DCB-A sample: (**a**) deposited carbon fiber beads with longitudinal furrows with lose broken fibers and matrix voids and (**b**) close-up with broken pulled-out fibers and plastic matrix deformation.

A fractographic analysis of the DCB-A specimens was carried out after the tests using SEM for a better understanding of the material behavior. As shown in Figure 15a, the fracture surface is irregular with clearly marked longitudinal furrows (darker and marked with red dashed lines) corresponding to the interspaces between fiber beads. These fiber beads are lighter and appear at a higher level in the image with irregular surfaces because of the individual fibers in them, some of them broken, and resin rich areas with what appear to be zones of open air bubbles or voids. It is also observed that carbon fibers are not straight but show some waviness inside the beads. Looking at the surface at a higher magnification, Figure 15b, many broken fibers pulled-out from the fiber filament accompanied by plastic matrix deformation are seen. This can be attributed to the effect of fiber-bridging during the test. Dry long undamaged fibers resulting from a lack of matrix impregnation are also observed.

**Figure 16:**
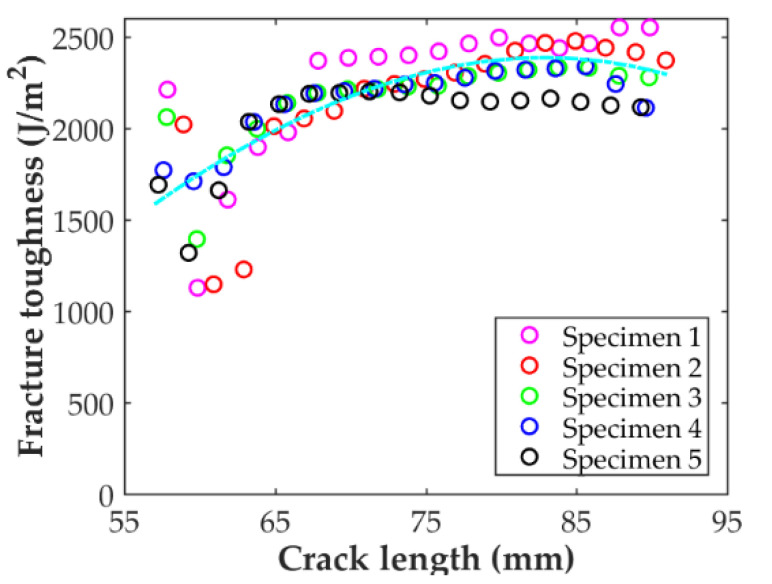
Crack propagation resistance curve for the DCB-A specimens calculated using the J-integral reduction method.

Good agreement between the ENF and C-ELS for *G*_II,ini_ is found when comparing the results for the interlaminar toughness in mode II summarized in Figure 10 and Table 4. Propagation values of *G*_II_ were only obtained with the C-ELS test. The R-curve for the C-ELS specimens is reported in Figure 16. After initiation, the curve increases up to a crack length of about 83 mm, where it starts to decrease slightly. It can be observed that the scatter for the first 3 mm of propagation is important, but it is greatly reduced after this point. Although the good match between the ENF and C-ELS values for the initiation value of *G*_II_, the R-curve for mode II seems to indicate that the initiation value could be slightly lower in C-ELS.

**Figure 17:**
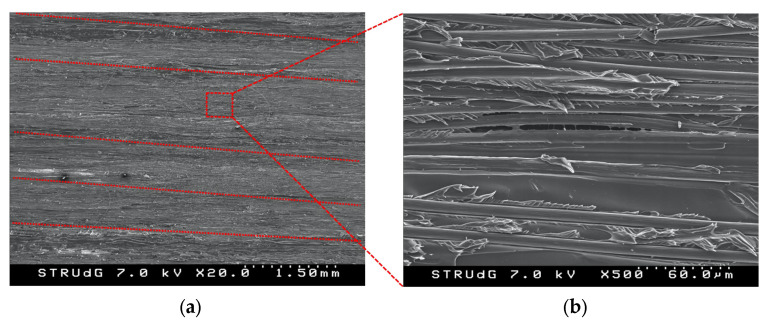
SEM images of a C-ELS fracture surface: (**a**) regular surface with deposited carbon fiber beads and longitudinal furrows and (**b**) close-up with evident matrix shear fracture.

A SEM fractographic analysis of the C-ELS specimens was also carried out after the tests to better understand the material behavior. Figure 17a shows the representative fracture surface of the C-ELS samples. In comparison to the fracture surface of the DCB specimen (Figure 15a), the mode II surface is smoother and the furrows between fiber beads are not so distinguishable (marked with red dashed lines). Again, it can be seen that fibers within the beads are not straight but display a certain waviness. However, very few broken or pulled-out fibers can be seen for the C-ELS case. This is due to the fact that the relative sliding between the two beams of the specimen does not generate the necessary stress-state to first pull-out the fibers from the matrix and then break them. Moreover, the frictional effect associated to this relative sliding helps to smooth the surface and make the furrows between fiber beads less evident. Matrix plastic deformation with inclined ridges due to shearing can be clearly observed when the mode II fracture surface is observed at a higher magnification, Figure 17b. There are matrix-dominated areas, zones with voids, dry fibers and fibers debonded from the surrounding matrix generated by a poor bonding interface.

The results found in this work show that both the initiation and propagation values of *G*_IIc_ are higher than the corresponding values of *G*_Ic_, both determined with the DCB-A and DCB-B specimens. This is an important remark because although it is the usual situation for most composite materials (*G*_IIc_ > *G*_Ic_), Iragi et al. [27] reported the opposite behavior for the same material. In their work, they justify the higher mode I fracture toughness based on a large number of fiber bridges during the mode I tests and the lack of matrix shearing during the mode II ones. However, analyzing their results in detail, it is seen that the reported value of the mode I fracture toughness, 2000 J/m^2^, corresponds to the value in the last point in their R-curve. However, the initiation in this curve is about 1600 J/m^2^, while the propagation value, taken as the mean value in the R-curve, would be about 1800 J/m^2^. These values are close to the ones obtained in the present study, 1497 J/m^2^ for initiation and 1720 J/m^2^ for propagation. Taking this into account, the value of the mode I fracture toughness reported by Iragi et al. is not so high when compared to the value of *G*_II_ they reported.

## 5. Conclusions

An experimental campaign has been carried out for characterizing the in-plane tensile and interlaminar mechanical properties of the CF/PA composite used in the 3D printer Markforged MarkTwo^®^. For the longitudinal tensile case, two different specimen geometries have been considered according to ASTM D638 and ASTM D3039 standards. The results show that the D638 fails prematurely due to stress concentration and the irregularity associated to the 3D printing start point in the fillet area (outside the gauge length), as similarly indicated by [5,14,15,29]. The in-plane longitudinal modulus and strength determined with the D3039 specimens and paper end-tabs are 16% and 42% higher, respectively. Thus, specimen D3039 is the best option to characterize the longitudinal properties of this type of material. Only the D638 geometry was considered for the in-plane transverse tensile characterization. It has been found that the fiber beads curvature or U-turns at the edges of the specimen, where part of the fiber becomes aligned with the applied load, results in higher in-plane transverse modulus and strength when compared with results present in the literature [27,34] using samples with full transverse fibers. Nevertheless, other works in the literature [35] report an in-plane transverse Young’s modulus almost twice the one determined in this work. Thus, it can be concluded that these edge effects caused by the fiber curvature at the edges can be reduced using wider specimens, such as the D3039, or eliminated if these edges are removed by cutting. Both the D638 and D3039 specimens have been used for the characterization of the in-plane tensile shear properties. In the D638 specimen the alignment of the fibers with the applied load was limited by the width of the specimen and the clamped area, resulting in a higher shear modulus and higher shear strength but lower strain at failure and plastic deformation—less than the 5% considered in the standard. On the other hand, the results obtained with the D3039 specimens are more consistent, with strains at failure of about 12.6% and similar tendencies to what has been reported by other authors.

The interlaminar fracture toughness in mode I and mode II of the Markforged CF/PA composite have been characterized. For this characterization, monolithic CF/PA specimens entirely manufactured using the 3D printer Markforged MarkTwo^®^ have been used without sandwiching them between two other composites or doublers. A procedure to use Kapton^®^ adhesive tape for generating the starter pre-crack during the printing process has been generated. For mode I, two different thicknesses have been considered for the DCB specimens to investigate any effect of the arm’s thickness on the results. It has been found that using 3 mm thick specimens, DCB-A, the propagation of the crack is smooth, not much fiber-bridging is involved, and the level of crack branching and jumping is also reduced in comparison to the case of 5 mm thick specimens, DCB-B. In fact, it has been observed that in the thicker specimens the crack propagated with a stick-slip response and with some large and unstable jumps, compromising the results. Thus, it can be concluded that the use of thick specimens is not appropriate for this interlaminar characterization of this material type. For mode II, both the ENF and C-ELS tests have been used, and it has been observed the initiation values are coincident for both types of tests. The propagation values and corresponding R-curve for mode II have been also obtained. Comparing the initiation and propagation values obtained for mode I and mode II, it has been observed that contrary to what has been already reported in the literature for this material, mode II fracture toughness values are higher than for mode I. The fractographic analysis of the delaminated surfaces showed that the printing process generates furrows between deposited fiber filaments, waviness in the fibers, resin rich areas, fibers with lack of impregnation or poor bonding between fiber and PA matrix and voids. In some cases it has been possible to observe what corresponds to fractured air bubbles. Comparing the typical surfaces for mode I and mode II, in mode I the furrows between fiber beads are more marked and more pulled-out, broken and lose fibers can be observed due to fiber-bridging during the test. In mode II, more matrix shearing is visible, which agrees well with the type of fracture expected for this type of test.

## Figures and Tables

**Table 1:** Configuration of the specimens for in-plane characterization of the material (N indicates nylon layer at 45 or −45°).

Tensile Test	ASTM Standard	Lay-Up	End Tabs	Main Dimensions (mm)
Longitudinal	D638	[N_2_/0_20_/N_2_]	---	165 × 13 × 3
	D3039	[N_1_/0_10_/N_1_]	Paper	250 × 15 × 1.5
Transverse	D638	[N_2_/90_20_/N_2_]	---	165 × 13 × 3
In-plane shear	D3518/D638	[N_2_/±45_5s_/N_2_]	---	165 × 13 × 3
	D3518/D3039	[N_1_/±45_4s_/N_1_]	Paper	250 × 25 × 2.5

**Table 2:** Configuration of the specimens for interlaminar fracture toughness characterization of the material (N indicates nylon layer at 45 or −45° and the location of the pre-crack). Width and thickness are once nylon cover has been removed.

Interlaminar Test	Standard	Lay-Up	Dimensions (mm)	Precrack (mm)
Mode I—DCB-A	ISO-15024	[N_4_/0_12_//0_12_/N_4_]	175 × 22.5 × 3	50
Mode I—DCB-B	ISO-15024	[N_2_/0_20_//0_20_/N_2_]	175 × 22.5 × 5	50
Mode II initiation	ASTM D7905	[N_1_/0_11_//0_11_/N_1_]	200 × 22.5 × 2.75	35
Mode II	ISO-15114	[N_1_/0_12_//0_12_/N_1_]	175 × 20 × 3	65

**Table 3:** Tensile in-plane mechanical properties of the CF/PA material (average value ± standard deviation).

	ASTM Standard	D638	D3039
Longitudinal	Tensile modulus (GPa)	57.1 ± 0.5	66.5 ± 7.1
	Poisson’s ratio	−	0.39 ± 0.03
	Tensile strength (MPa)	528 ± 27.2	752 ± 88.6
	Ultimate tensile strain (%)	0.85 ± 0.05	1.10 ± 0.08
Transverse	Tensile modulus (GPa)	6.16 ± 1.1	−
	Tensile strength (MPa)	49.3 ± 10	−
	Ultimate tensile strain (%)	0.93 ± 0.07	−
	**ASTM Standard**	**D3518/D638**	**D3518/D3039**
In-plane shear	Shear modulus (GPa)	4.87 ± 0.86	2.13 ± 0.17
	Shear stress at 5% (MPa)	−	49.0 ± 2.0
	Maximum shear stress (MPa)	88.7 ± 4.9	56.9 ± 1.7
	Maximum shear strain (%)	2.49 ± 0.16	12.6 ± 2.3

**Table 4:** Initiation and propagation mode I and mode II interlaminar fracture toughness values for the CF/PA material.

		Fracture Toughness (J/m^2^)	
Specimen	Data Reduction	Initiation	Propagation
DCB-A	J-integral	1497 ± 85	1720 ± 116
DCB-B	J-integral	1064 ± 125	1265 ± 57
ENF	CC	1991 ± 80	−
C-ELS	CBTE	1950 ± 215	2307 ± 117

**Table 5:** Comparison between the mechanical properties of the CF/PA composite reported in the literature and the ones found in this work. The two values indicated for *E*_22_ and *Y*^t^ in Todoroki et al. [34] correspond to specimens without and with fiber U-turns at the edges. (* Values are inferred from the data reported by Pyl et al. [29] for a single specimen).

Reference	*E*_11_ (Gpa)	*E*_22_ (Gpa)	*G*_12_ (Gpa)	*X*^t^ (Mpa)	*Y*^t^ (Mpa)	*S* (Mpa)
Blok et al. [31]	62.5	−	2.3	986	−	31
Justo et al. [32]	68	−	−	701	−	−
Pyl et al. [29]	58	1.5/1.8 *	4	719	40 *	48
Chabaud et al. [33]	60	−	−	534	−	−
Iragi et al. [27]	69.4	3.5	1.9	905	17.9	43
Todoroki et al. [34]	61	4/5	2.1	701	19/25	52
Yogeshvaran et al. [35]	54	12	5	860	19	25
This work	66.5	6.2	2.1	752	49.3	49

## Data Availability

Data is contained within the article.
